# Fostering and sustaining collaborative innovation: Insights from ELIXIR Europe's life science Communities

**DOI:** 10.12688/f1000research.168288.1

**Published:** 2025-09-09

**Authors:** Clare Garrard, Katharina F Heil, Maria Cristina Aspromonte, Bérénice Batut, Magda Chegkazi, John M Hancock, Elaine Harrison, Naveed Ishaque, Giselle Kerry, Eija Korpelainen, Jerry Lanfear, Corinne Martin, Sebastian Schaaf, Serena Scollen, Yun-Yun Tseng, Sameer Velankar, Juan Antonio Vizcaíno, Robert M Waterhouse, Egon Willighagen, Niklas Blomberg, Peter Maccallum

**Affiliations:** 1 ELIXIR, Wellcome Genome Campus, Hinxton, Cambridge, UK; 2 Department of Biomedical Sciences, University of Padova, Padova, Italy; 3 IFB-core, IFB, CNRS, INSERM, INRAE, CEA, Institut Français de Bioinformatique, Villejuif, 94800, France; 4 Université Clermont Auvergne, AuBi, Mésocentre Clermont-Auvergne, Aubiere, France; 5 Institute of Biochemistry, Faculty of Medicine, University of Ljubljana, Ljubljana, Slovenia; 6 Berlin Institute of Health, Center of Digital Health, Charité - Universitätsmedizin Berlin, Berlin, Germany; 7 CSC - IT Center for Science, Espoo, Finland; 8 Galaxy Europe, Freiburg Team, Bioinformatics Group, Department of Computer Science, University of Freiburg, Freiburg, Germany; 9 EMBL-EBI, Wellcome Trust Genome Campus, European Molecular Biology Laboratory, European Bioinformatics Institute, Hinxton, Cambridge, UK; 10 Quartier Sorge, Batiment Amphipole, SIB Swiss Institute of Bioinformatics, Lausanne, 1015, Switzerland; 11 Department of Bioinformatics BiGCaT, NUTRIM, Maastricht University, Maastricht, The Netherlands

**Keywords:** Community, Communities, Life Science, Community Management, Research Infrastructure, Community of practice, Bioinformatics Services, Data

## Abstract

Communities of experts collaborating on scientific or technical projects are drivers of innovation across the life sciences. The ELIXIR research infrastructure organises scientific- and technological-themed communities as one of its key mechanisms to ensure that services are user-focused, while at the same time facilitating collaboration and creating scientific impact through the life science data generated across Europe. ELIXIR has rapidly expanded its communities portfolio in response to unmet needs and has developed a comprehensive process framework to facilitate the work of these communities.

The ELIXIR Communities framework is made up of a suite of tools and processes that ensure effective community evolution and management, covering how communities are established, led, supported, and can collaborate across ELIXIR and beyond. Being aware of similar approaches in other contexts and in the interests of furthering community development in other research infrastructures and similar organisations, we share insights into the ELIXIR Communities framework and outline the skill set of a community manager and what this looks like in the ELIXIR context.

Finally, to show the benefits of the communities, we share concrete examples of how the ELIXIR Communities have had an impact on the scientific landscape. By showcasing these outcomes we hope to demonstrate not only to other research infrastructures, but also to funders, that supporting scientific communities provides a valuable return on investment. We hope that these examples will encourage life scientists who may be interested in joining the ELIXIR Communities, and research infrastructure professionals whose roles require structured engagement with domain experts and users.

## Introduction

Scientific communities play a pivotal role in facilitating effective collaboration and feedback to technology providers in research infrastructures such as ELIXIR Europe ( https://elixir-europe.org/). The significance of such collaborative efforts has notably increased over time, yielding highly impactful datasets and publications. ^1, 2^ Over the ten years since it became operational in 2014, ELIXIR has created a formalised framework for communities, and as of its ten-year anniversary (December 2023) boasts seventeen scientific communities made up of technical experts from across Europe and beyond ( https://elixir-europe.org/communities). The purpose of ELIXIR Communities is to capture user needs into formal requirements and drive the development and uptake of bioinformatics services (such as databases, software, cloud computing, standards and training) by providing use cases in strategically important application areas. The creation of such formalised communities, coupled with sustained support, ensures ongoing momentum and drives far-reaching impact.

This article offers a playbook for other research infrastructures or similar initiatives aiming to develop a comparable community system to ensure user-led service provision. Recognising that sustainability and funding hinges on outcomes, we aim to showcase some of the impacts and other successes of the ELIXIR Communities. Finally, we offer insight to technical experts who are interested in exploring the ELIXIR Communities or exploring the career path of community management.

## Evolution of ELIXIR communities

The ELIXIR Europe research infrastructure was founded in December 2013, its mission being the ‘construction and operation of a sustainable infrastructure for biological information in Europe to support life science research and its translation to medicine and the environment, the bio-industries and society’. ^3^ Driven by its extensive people network, that as of December 2023, spans 24 countries (21 members and 3 observers) and the European Molecular Biology Laboratory (EMBL), ELIXIR has made substantial progress in this mission, and the ELIXIR Communities have been one of the key driving forces.

The ELIXIR Communities evolved out of four initial use cases (Human Data, Rare Diseases, Marine Metagenomics, and Plant Sciences) that were initiated in the ELIXIR-coordinated EU-funded project, ELIXIR-EXCELERATE. ^4^ The ELIXIR-EXCELERATE project was the first major project in ELIXIR aimed at building the operational structures so that ELIXIR services, provided via national Nodes, could be better coordinated and further developed. The use cases within this project addressed the challenge of ensuring that technological service offerings would serve the diverse needs of life scientists and create alignment on technical standards across Europe. Following the end of the project the ELIXIR Communities framework was developed as a mechanism to sustain these collaborative efforts.

Today, communities allow ELIXIR to interact with a broad set of initiatives and projects, including other research infrastructures (e.g. through cluster projects such as EOSC-life) and strategic policy initiatives such as ones emerging from and sustained by the European Strategy Forum on Research Infrastructures (ESFRI) ( Table 1). The ELIXIR Communities also collaborate with one another, particularly where their scientific areas align, such as the Intrinsically Disordered Proteins (IDP), Proteomics and 3D-BioInfo Communities since all work on protein research, and the Federated Human Data, Rare Disease and human Copy Number Variation (hCNV) Communities, which support the discovery, access, sharing and analysis of human genomic and linked data on a massive scale.

**Table 1.** Examples of links between ELIXIR Communities and projects, other research infrastructures, and global initiatives.

ELIXIR community	Links to projects, initiatives and other research infrastructures
3D-BioInfo	Instruct-ERIC ( https://instruct-eric.org/) 3D-Sig Structural Bioinformatics Community of the International Society for Computational Biology (ISCB, https://www.iscb.org/)
Biodiversity	BGE ( https://biodiversitygenomics.eu/) - Biodiversity Genomics Europe BiCIKL ( https://bicikl-project.eu/) - Biodiversity Community Integrated Knowledge Hub COL ( https://www.catalogueoflife.org/) - the Catalogue of Life ERGA ( https://www.erga-biodiversity.eu/) - the European Reference Genome Atlas, European node of the Earth BioGenome Project GBIF ( https://www.gbif.org/) - Global Biodiversity Information Facility iBOL EUROPE ( https://iboleurope.org/) - European node of the International Barcode of Life LifeWatch-ERIC ( https://www.lifewatch.eu/) - the European e-Science infrastructure for biodiversity and ecosystem research NFDI4Biodiversity ( https://www.nfdi4biodiversity.org/en/who-we-are/) - German National Research Data Infrastructure for biodiversity data
Federated Human Data	B1MG ( https://b1mg-project.eu/) BBMRI-ERIC ( https://www.bbmri-eric.eu/) Beacon ( https://beacon-project.io/) BY-COVID ( https://by-covid.org/) CINECA* ( https://cordis.europa.eu/project/id/825775) FEGA ( https://ega-archive.org/about/projects-and-funders/federated-ega/) - Federated European Genome-Phenome Archive ELIXIR-CONVERGE* ( https://cordis.europa.eu/project/id/871075) European Genomic Data Infrastructure (GDI, https://gdi.onemilliongenomes.eu/) GA4GH ( https://www.ga4gh.org/) - the Global Alliance for Genomics & Health
Food & Nutrition	NuGO ( https://www.nugo.org/) - the Association of Universities and Research Institutes focusing on the joint development of the research area of molecular nutrition, personalised nutrition, nutrigenomics and nutritional systems biology PIMENTO COST Action ( https://fermentedfoods.eu/)
Galaxy	AgroServ ( https://agroserv.eu/) Australian BioCommons ( https://www.biocommons.org.au/) BGE ( https://biodiversitygenomics.eu/) - Biodiversity Genomics Europe BIO Network for Training (BioNT, https://biont-training.eu/), EOSC EuroScienceGateway ( https://www.eurosciencegateway.org) Euro-Bioimaging ( https://www.eurobioimaging.eu/) European Galaxy community ( https://galaxyproject.org/eu/) European Genomic Data Infrastructure (GDI, https://gdi.onemilliongenomes.eu/) European Grid Infrastructure (EGI, https://www.egi.eu/about/) GA4GH ( https://www.ga4gh.org/) - the Global Alliance for Genomics & Health Gallantries project ( https://gallantries.github.io/) German National Research Data Infrastructures (NFDI DataPLANT, NFDI4BIOIMAGE) German Network for Bioinformatics Infrastructure (de. NBI, https://www.denbi.de/) Global Galaxy Project community ( https://galaxyproject.org/) Other EOSC (EOSC-Pillar, EOSC Nordic, BY-COVID, EOSC-Life, EOSC4Cancer, AquaINFRA, FAIR-EASE, Skills4EOSC, OSCARS) Vertebrate Genome Project (VGP, https://vertebrategenomesproject.org/)
Human Copy Number Variation (hCNV)	Beacon ( https://beacon-project.io/) GA4GH ( https://www.ga4gh.org/) - the Global Alliance for Genomics & Health
Intrinsically Disordered Proteins (IDP)	Bioschemas ( https://bioschemas.org/) CAID Initiatives ( https://caid.idpcentral.org/) Gene Ontology (GO) Consortium ( https://geneontology.org/) Evidence and Conclusion Ontology (ECO) ( https://evidenceontology.org/) HUPO-Proteomics Standards Initiative ( https://www.psidev.info/) IDP Central Consortium ( https://idpcentral.org/) IDPfun international consortium ( https://idpfun.eu/) InterPro Consortium ( https://www.ebi.ac.uk/interpro/about/consortium/) Non-globular proteins in the era of Machine Learning (ML4NGP COST Action, https://ml4ngp.eu/) PhaseAge project ( https://phasage.eu/)
Marine Metagenomics	Genome Standards Consortium ( http://www.gensc.org/) LifeWatch-ERIC ( https://www.lifewatch.eu/) - the e-Science European infrastructure for biodiversity and ecosystem research Metaproteomics Initiative ( https://www.metaproteomics.org/)
Metabolomics	Bioschemas ( https://bioschemas.org/) PhenoMeNal project* ( https://cordis.europa.eu/project/id/654241)
Microbial Biotechnology	IBISBA ( https://www.ibisba.eu/) - Industrial Biotechnology Innovation and Synthetic Biology Accelerator
Microbiome	MicrobiomeSupport ( https://www.microbiomesupport.eu/) National Microbiome Data Collaborative ( https://microbiomedata.org/)
Plant Sciences	AnaEE ( https://www.anaee.eu/) - Analysis and Experimentation on Ecosystems DataPLANT ( https://nfdi4plants.github.io/) - German national research data infrastructure for Plant Sciences EMPHASIS ( https://emphasis.plant-phenotyping.eu/) - European Infrastructure for Plant Phenotyping FAIRagro ( https://fairagro.net/en/) - German national research data infrastructure for Agrosystems
Proteomics	EuBIC-MS community ( https://eubic-ms.org/) HUPO-Proteomics Standards Initiative (PSI, https://www.psidev.info/) ProteomeXchange Consortium of proteomics resources ( https://www.proteomexchange.org/)
Rare Diseases	BBMRI-ERIC ( https://www.bbmri-eric.eu/) EATRIS ( https://eatris.eu/) ECRIN ( https://ecrin.org/) EJP RD ( https://www.ejprarediseases.org/) - European Joint Programme on Rare Diseases FAIR-dICT* ( https://www.dtls.nl/fair-data/fair-dict/) GA4GH ( https://www.ga4gh.org/) - the Global Alliance for Genomics & Health ODEX4ALL* ( https://research-software-directory.org/projects/odex4all) Orphanet consortium ( https://www.orpha.net/) RD-Connect* ( https://rd-connect.eu/)
Research Data Management	ELIXIR-CONVERGE* ( https://cordis.europa.eu/project/id/871075) Research Data Alliance - Life Science Data Infrastructures Interest Group ( https://www.rd-alliance.org/groups/life-science-data-infrastructures-ig)
Single-Cell Omics	Global Alliance for Spatial Technologies (GESTALT) Global Organisation for Bioinformatics Learning, Education and Training (GOBLET, https://www.mygoblet.org/training-portal/) Human Cell Atlas (HCA, https://www.humancellatlas.org/)
Systems Biology	COMBINE ( https://co.mbine.org/) Disease Maps Consortium ( https://disease-maps.io)
Toxicology	Bioschemas ( https://bioschemas.org/) NanoSafety Cluster ( https://www.nanosafetycluster.eu/) NORMAN Network ( https://www.norman-network.net/) Partnership for the Assessment of Risk from Chemicals (PARC, https://www.eu-parc.eu/) USA EPA CompTox Dashboard ( https://comptox.epa.gov/dashboard/) USA NIH PubChem ( https://pubchem.ncbi.nlm.nih.gov/)

Table showing ELIXIR Communities and links to projects, initiatives and other research infrastructures. Completed projects are marked with a *.

Additionally, Community members can be part of more than one research infrastructure. Overlaps happen especially within the scope of Life Science research infrastructures ( https://lifescience-ri.eu/).

## ELIXIR communities framework

The ELIXIR Communities framework provides a formalised structure through which support can be channelled to important scientific areas. To develop and sustain these communities and support their continued impact, ELIXIR has developed several tools and processes.

### The community establishment process

**Figure 1.**
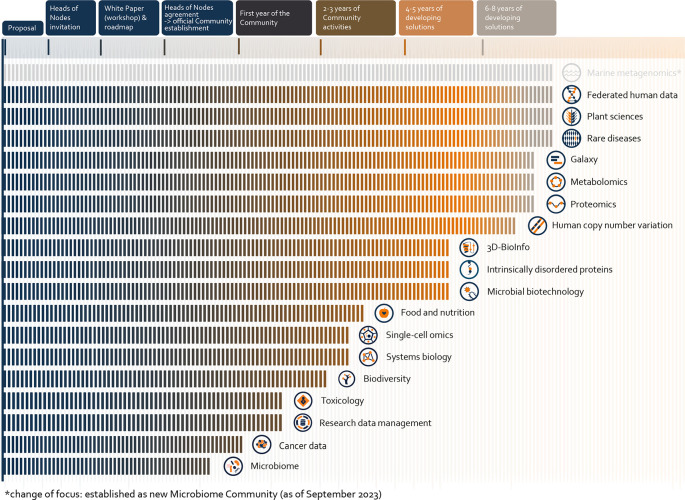
Stages in the development of ELIXIR Communities. Timeline to show the progression of each ELIXIR Community through the stages of Community maturity, following the processes outlined along the top row. Once a community has reached the phase of Heads of Nodes approval (i.e. approval by the country-level bioinformatics leads), it becomes an official ELIXIR Community and can begin work on an internal project funded through ELIXIR’s core funds. The most mature Communities are shown in the top right (these were the four ELIXIR-EXCELERATE Use Cases), with the newest Communities shown in the bottom left (Communities portfolio as of December 2023).

ELIXIR Communities follow a defined path to maturity, which is formally embedded in ELIXIR’s processes as a treaty-based intergovernmental organisation, and sets them up for self-organisation and collaboration ( Figure 1). Importantly, the majority of ELIXIR Communities that exist today were formed through a bottom-up approach, which ensures broad grass-roots support and interest.

The first step for a prospective ELIXIR Community is for an initial group of interested parties to put forward a proposal highlighting the relevance and impact of the scientific area, as well as demonstrating broad benefit and buy-in across the ELIXIR country-level “Nodes” and beyond. The group will then give a formal presentation to the Heads of Nodes governance body of ELIXIR, which is composed of national bioinformatics representatives. Following consultation and approval, the group is invited to write a white paper outlining their community goals and strategic position in the scientific landscape. Examples of such white papers can be found in Table 2. The white paper is circulated for feedback, and finally, the Heads of Nodes are formally consulted on the white paper. At this point, the ELIXIR Community may be formally established and become eligible to receive ELIXIR support (such as funding through internal projects, or for knowledge exchanges) and participate in ELIXIR events and projects as an ELIXIR Community. They will then begin working towards the Community goals and represent their specific scientific domain within the ELIXIR research infrastructure. This process has evolved and matured over time in parallel with the formalisation and growth of the communities portfolio.

**Table 2.** ELIXIR community white papers.

ELIXIR community	White paper
3D-BioInfo	A community proposal to integrate structural bioinformatics activities in ELIXIR (3D-Bioinfo Community) ^5^
Biodiversity	The ELIXIR Biodiversity Community: Understanding short- and long-term changes in biodiversity ^6^
Federated Human Data	ELIXIR EXCELERATE use case ^4^
Food & Nutrition	The future of food and nutrition in ELIXIR ^7^
Human Copy Number Variation	The ELIXIR Human Copy Number Variations Community: building bioinformatics infrastructure for research ^8^
Intrinsically Disordered Proteins	An intrinsically disordered proteins community for ELIXIR ^9^
Marine Metagenomics	ELIXIR EXCELERATE use case ^4^ ELIXIR pilot action: Marine metagenomics – towards a domain specific set of sustainable services ^10^
Metabolomics	The future of metabolomics in ELIXIR ^11^
Microbiome	Establishing the ELIXIR Microbiome Community ^12^
Plant Sciences	ELIXIR EXCELERATE use case ^4^ Roadmap: ELIXIR Plant sciences 2020-2023 Roadmap ^13^
Proteomics	A community proposal to integrate proteomics activities in ELIXIR ^14^
Rare Diseases	ELIXIR EXCELERATE use case ^4^
Research Data Management	A research data management (RDM) community for ELIXIR ^15^
Single-Cell Omics	Community-driven ELIXIR activities in single-cell omics ^16^
Systems Biology	Systems Biology in ELIXIR: modelling in the spotlight ^17^
Toxicology	ELIXIR and Toxicology: a community in development ^18^

The community's establishment process helps its members to clarify their roles, responsibilities and overarching goals at an early stage and plays an important role in shaping the identity and function of the community. Notably, writing a white paper together and then being able to work towards these goals through an internal project, funded by ELIXIR’s core funds, offers an opportunity for community members to work together and become a cohesive group. The benefits of this process are also reflected in the literature, where having goals for the community and opportunities for collaborative participation have been highlighted as factors for community success. ^2^

### Networking opportunities and the human element

In any community, a sense of belonging is what keeps people involved. ^19, 20^ Members of ELIXIR Communities have the opportunity to meet other experts, exchange ideas, and do meaningful work in their area of interest through project work. As part of this, community members build social capital, and gain leadership experience, e.g., through leading work packages, which helps their professional advancement.

ELIXIR supports this by providing and facilitating opportunities for connection, collaboration, and sharing ongoing work, such as at monthly virtual Community meetings, and annual events such as the ELIXIR All Hands Meetings, Community Face-to-Face meetings, and BioHackathon Europe. ^21^ These events offer opportunities for people to work together on scientific literature, tools, services, standards, and training, at the same time building social capital. For example, ELIXIR Communities form a key part of BioHackathon Europe and its projects, and the event has been reported by participants to broaden one’s professional network. ^21^

Proactive measures can also be taken to promote inclusion and thus belonging, such as the creation of psychological safety, upholding respect for others as a key value, and involving diverse members in the working group. ^22^ Within ELIXIR, there are several mechanisms to support this including an Equality, Diversity and Inclusion working group at the ELIXIR Hub, an Equal Opportunity Strategy, ^23^ and ELIXIR’s Code of Conduct ( https://elixir-europe.org/events/code-of-conduct) which aims to foster a culture of respect within the network, such as by having Code of Conduct allies at every ELIXIR event. Rewardingly, the Code of Conduct has also been used as a starting point for other organisations that are part of the ELIXIR network to begin to develop their own Codes of Conduct.

Additionally, ELIXIR organises outreach to increase the visibility and profile of its Communities, through events such as ELIXIR Webinars, the ELIXIR Communities track at the bi-annual European Conference on Computational Biology (ECCB) e.g., ECCB 2022 ( https://elixir-europe.org/events/elixir-eccb-2022) and ECCB 2024 ( https://elixir-europe.org/events/elixir-eccb-2024), and support for the joint ISMB/ECCB meeting in alternating years.

### Co-leads and governance

ELIXIR ensures scientific leadership of each ELIXIR Community through its Community co-leads, who guide the scientific direction of the Community. This includes driving activities to fulfil the Community goals, engaging in projects related to the Community, and evolving the vision of the Community over time.

We conducted a survey of the ELIXIR Community co-leads in the fourth quarter of 2023 to assess the impact of having this role. The survey used a combination of open-ended questions and ranked questions using the Likert scale, from strongly agree to strongly disagree. ^24^ Out of 22 respondents (representing 42% of the overall co-lead group), 82% of co-leads agreed or strongly agreed that the position helped them to make an impact in their chosen field. Scientific impact was widely recognised as the main area of impact, with examples such as coordination of efforts through community building, and the ability to drive technological developments and strategic direction. Co-leads also described having more confidence in the success of grant proposals where they were able to cite their community’s involvement in the proposed activities.

The Community co-lead position was recognised as an opportunity for career development. Almost half (46%) of co-leads felt that the role had helped advance their career development or provided professional opportunities, with most of the remainder (41%) giving a neutral answer. While the survey was kept anonymous, based on our insights on the co-leads career stages we suggest that these values reflect the range of career stages of the co-leads and the possible benefits that can come with the role. It is likely that more established co-leads do not necessarily need the same level of career development, if any (in the case of senior co-leads), but still benefit from other aspects of the role. A common theme of the benefits of the co-lead role for visibility and profile was clear from the survey results and was cited as influencing co-lead promotion prospects.

### The communities handbook

The Communities Handbook ^25^ is a reference tool that Community members use to understand various processes and day-to-day activities of an ELIXIR Community. This includes topics such as Community purpose and structure, benefits and impact, as well as eligibility criteria for joining an ELIXIR Community. These processes contribute to the sense of community and aim to facilitate the scientific work within the Community.

### Community management

Each ELIXIR Community receives direct support from one designated staff member at the ELIXIR Hub, which is the coordinating secretariat of the distributed infrastructure, currently referred to as “Community liaison”. Although ELIXIR does not explicitly use the job title “Community Manager”, the skills portfolio required for people who support the Communities overlaps broadly with the skills for community managers laid out by the Center for Scientific Collaboration and Community Engagement ( https://www.cscce.org/). These skills fall into five broad categories: technical, interpersonal, communication, programme management, and programme development. ^26^

As an illustration of this skills portfolio, staff supporting ELIXIR Communities have a high level of technical expertise, having attained an advanced scientific degree and/or worked in a technical role themselves before moving into a coordination role. This includes domain-specific expertise and hands-on experience, a combination vital to effective communication with community members. Secondly, since much of the work of ELIXIR Community management is facilitating collaboration across Europe, interpersonal skills such as cultural competence, engagement, and consultation are central to the role. Thirdly, to support the ELIXIR Communities effectively, there is a need for communication skills such as presenting, content creation, and knowledge brokering to foster connections between different groups. Finally, programme development and programme management skills are needed to successfully design, adapt, and implement the ELIXIR Communities programme. This spans the skillset from strategy development to the day-to-day time management, event planning, and meeting facilitation that keeps the communities running smoothly.

### Portfolio management and monitoring community progress

As part of managing the portfolio of ELIXIR Communities, ELIXIR tracks Community activities and successes, including the areas where a Community might be facing challenges. This is monitored continuously, as well as in formal reviews by ELIXIR technical experts and governance bodies such as the Heads of Nodes and the Scientific Advisory Board.

Every few years, individual ELIXIR Communities are reviewed based on factors such as their leadership and membership, level of interconnectedness within the ELIXIR ecosystem, and whether the Community is meeting the goals initially proposed in its white paper. The Communities review form shows all currently established review criteria. ^27^ This review process is a helpful milestone for reflection and feedback and has assisted the Communities in maturing further.

The most active ELIXIR Communities have naturally created many useful and impactful outputs. However, some communities may become dormant due to various constraints such as time or funding, and others change their focus to better serve their members’ interests. For example, the established ELIXIR Marine Metagenomics Community has become dormant as its leads and members have developed a new Microbiome Community of similar but wider scope. This highlights the need to continually reshape the Communities portfolio to adapt to a changing research landscape.

## ELIXIR community case studies

The themes of ELIXIR Community impacts take various forms - whether it is improving research efficiency by centralising data, contributing to a wider effort, or addressing gaps with much-needed community standards and domain-specific training.

### Organising and FAIRifying biological data

The ELIXIR 3D-BioInfo Community in Structural Bioinformatics ( https://elixir-europe.org/communities/3d-bioinfo) developed the Protein Data Bank in Europe - Knowledge Base (PDBe-KB; https://pdbe-kb.org) initially with BBSRC (UK) funding but subsequently expanded through ELIXIR project funding. It is an open, collaborative consortium for integrating 3D-structure data and functional annotations to enable basic and translational research. More than 30 research groups contribute to the PDBe-KB to increase the visibility and reduce the fragmentation of 3D-structure annotations available in specialist partner data resources and make these data findable, accessible, interoperable and reusable (FAIR). ^28^ The aim is to place macromolecular structure data in their biological context, to facilitate basic and translational research. The 3D-Beacons Network ( https://3dbeacons.org), which is also enabled through a combination of ELIXIR and BBSRC funding, is integral to the PDBe-KB consortium activities, providing integrated access to experimental and predicted protein structure models and meta-information in a standardised data format.

### Contributions to global efforts

The Galaxy project began at the Pennsylvania State University in 2006 and has since grown to become a global effort. The European Galaxy Server ( https://usegalaxy.eu/), which underpins the ELIXIR Galaxy Community ( https://elixir-europe.org/communities/galaxy), is one of just three major ‘usegalaxy’ instances supported by the NIH and the US National Science Foundation - the other two being in the US and Australia. In addition there are multiple growing international efforts, e.g., in India ( https://www.galaxyproject.in), Africa ( https://africa.usegalaxy.eu/) and recently Canada. The European Galaxy Server alone has had more than 90,000 users since November 2018 ^29^ and has been cited in 547 publications between 2017 and 2023, ^30^ illustrating the wide-reaching impact of Galaxy on improving research efficiency.

The community receives national funding through the German Federal Ministry of Education and Research, and de. NBI (the German Node of ELIXIR; https://www.denbi.de/), which has been key in building and operating the European Galaxy Server infrastructure. The ELIXIR Galaxy Community has additionally created connections and facilitated knowledge exchange across borders - both within Europe, such as a knowledge exchange between the German Node and visiting researchers from Italy and Estonia, and beyond, such as the ELIXIR-funded staff exchange with Australia in 2023 ( https://elixir-europe.org/news/australia_visit_2023 ).

### Establishing community standards

A key challenge that is addressed by many ELIXIR Communities is the lack of community standards for different life science domains. A well-known example of this is the Minimum Information About Plant Phenotyping Experiments (MIAPPE) ^31^ and Breeding API (Application Programming Interface) ^32^ data standards that were developed through the ELIXIR Plant Sciences Community ( https://elixir-europe.org/communities/plant-sciences ).

Another key example is the role that the Federated Human Data Community ( https://elixir-europe.org/communities/human-data ), the Human Copy Number Variation (hCNV) Community ( https://elixir-europe.org/communities/human-copy-number-variation ), and the Rare Disease Community ( https://elixir-europe.org/communities/rare-diseases ) have played in driving genomic standards development through their work with GA4GH ( https://www.ga4gh.org) in an effort to prevent silos in data sharing and ensuring that standards are endorsed by the wider scientific genomic community. One prominent example is Beacon V2 ( https://docs.genomebeacons.org/), which enables the discovery of genomic variants and associated information without jeopardising the privacy of the dataset, and is being implemented in the European Genomic Data Infrastructure ( https://gdi.onemilliongenomes.eu/) and more recently, the Federated EGA ( https://ega-archive.github.io/FEGA-onboarding/). Interestingly, the ELIXIR Plant Sciences Community has developed extensions of the Beacon standards to make plant datasets discoverable, demonstrating further value of ELIXIR via shared developments across domains.

In the area of protein research, the IDP Community has developed the Minimum Information About Disorder Experiments (MIADE) guidelines ^33^ in collaboration with the HUPO-PSI initiative ( https://www.psidev.info/), to maximise the interpretation and dissemination of IDP experiments. Similarly, the Proteomics Community created a metadata standard format for capturing the experimental design information in public datasets, the Sample and Data Relationship File (SRDF)-Proteomics format, ^34^ in collaboration with the EuBIC-MS community ( https://eubic-ms.org/) and with HUPO-PSI. Three years after the first version of SDRF-Proteomics, the format is increasingly used and different tools are being developed supporting it, ^35^ including as a key point, the developments in the PRIDE database ^36^ to improve the metadata annotation of public datasets.

### Finding and addressing user needs & training gaps

A common thread in the ELIXIR Communities is the goal to improve training within their domain. The Single-Cell Omics ( https://elixir-europe.org/communities/single-cell-omics) and Systems Biology Communities ( https://elixir-europe.org/communities/systems-biology ) are good examples of this, having each recently done a landscape survey of the training available in their field. They are currently working towards addressing the identified training gaps.

The creation of the Single-Cell Omics Community was largely driven by the demand from researchers for training in the rapidly developing single-cell and spatial technologies which have been accompanied by the active development of data analysis methods. This was coupled with the Europe-wide network of bioinformatics training providers seeing the benefits of working together through the ELIXIR framework. This demonstrates the crucial role that the grassroots, requirement-driven aspect of community development plays in curating the ‘right’ portfolio of ELIXIR Communities. Notably, dealing with the challenges in benchmarking, training and data standards is simply not possible without international collaboration.

### Community similarities and distinctions

Communities are a common way of organising people in science and beyond. ^19^ Some, like the ELIXIR Communities, are aligned with specific products or infrastructures, while others place emphasis on simply fostering communities of practice for like-minded people to come together. In both types of community, there is a common thread of shared goals and interests.

Initiatives like The Carpentries ( https://carpentries.org/community/) and WILDLABS ( https://wildlabs.net/) exemplify the philosophy of a community of practice. The Carpentries works to build both local and global communities of practice to build capacity in data analysis, computational thinking and research software development. Similarly, the WILDLABS community brings together conservation technology users and makers who want to find solutions to the many challenges facing nature today.

Similarly, communities that centre around an infrastructure or product also have an aspect of acting as a community of practice that brings together like-minded individuals who network and share knowledge, but in addition to this, they enhance the quality and relevance of the resources they are linked to. For example, the Australian BioCommons operates across diverse domains ( https://www.biocommons.org.au/domains), facilitating communities geared towards fostering discussion and learning, along with identifying digital infrastructure gaps. This mirrors the ELIXIR Communities' function as a conduit for feedback. Additionally, communities such as Galaxy's Special Interest Groups ( https://galaxyproject.org/community/sig) and GA4GH's Communities of Interest ( https://www.ga4gh.org/what-we-do/communities-of-interest/) drive tailored solutions within their respective domains while pinpointing gaps in existing offerings.

## Discussion & conclusion

The ELIXIR Communities are an integral part of the ELIXIR infrastructure, connecting technology providers to domain experts across Europe. The cross-border nature of ELIXIR Communities contributes to a harmonised approach which is vital to providing a concerted and reliable service offering. By connecting with wider initiatives ( Table 1), the ELIXIR Communities bridge gaps between multiple projects and initiatives, thus facilitating knowledge sharing, accelerating technology development, and unlocking new and attractive funding opportunities. This highlights the important role that a research infrastructure such as ELIXIR can play in fostering collaborations not only through funding, but also through cross-community networking.

Benefits for ELIXIR Community members include expanding their network, gaining exposure to larger initiatives and peer recognition, and opportunities for growth and skills development through participation in new projects. The effect of broadening one’s network and exposure to new opportunities is true for any community of practice, and its importance for professional growth should not be underestimated.

Thus, the ELIXIR Communities not only provide benefits to the research infrastructure, Community members and the wider scientific community, but also serve as an effective way for the research infrastructure to ensure user-centric service provision. This principle holds for any organisation developing products or services for a wider community.

Additionally, the Communities benefit the Nodes and institutes in the ELIXIR network by offering a valuable opportunity to grow collaborations and visibility, which is especially important for countries with less advanced scientific infrastructure. Through tools and processes highlighted in this article - such as a handbook to guide Communities, co-leads to champion communities, and community managers to facilitate communities - we hope to offer a starting point for other organisations looking to initiate or improve similar groups.

Being a pan-european research infrastructure, ELIXIR has the opportunity, motivation, and expertise to develop and support communities that align with the EU’s Horizon Europe priority areas. This brings with it the challenge of ensuring that there are enough scientists involved in the Communities to maintain vital grassroots support. An expanding portfolio of Communities also brings with it organisational challenges: a larger Communities portfolio would entail more Community managers, and thus it may be worth exploring partnerships between research infrastructures to lower the cost of community management and forge stronger links across the scientific community.

Community management plays an important role in the success of ELIXIR’s Communities portfolio. While organisations such as the CSCCE have taken important steps in supporting community managers and recognising their contributions, this also represents an opportunity for ELIXIR and other scientific organisations that leverage communities to be involved in promoting the role of a community manager, to make it more appealing for researchers to explore this less traditional but valuable science career path.

In conclusion, the far-reaching impacts of ELIXIR Communities are demonstrable. From their crucial role in ensuring user-centric, fit-for-purpose resources and improving alignment across Europe and beyond, to their tangible impacts such as organising centralised data or creating training initiatives, the ELIXIR Communities demonstrate their influence in the scientific world. Beyond the immediate benefits of these impacts lies a strategic complementarity: not only do these communities deliver value, but they also ensure that funders recognise the significance of their investments. This recognition positions impactful groups for sustained support in future projects, reaffirming the enduring value of community-driven structures in scientific endeavours, which will continue to benefit science and society in the long term.

## Underlying data

No data are associated with this article.

## References

[1] Wuchty S Jones BF Uzzi B : The increasing dominance of teams in production of knowledge. *Science (1979).* 2007 May 18 [cited 2024 Mar 3];316(5827):1036–1039. 10.1126/science.1136099 17431139

[2] Budd A Corpas M Brazas MD : A Quick Guide for Building a Successful Bioinformatics Community. *PLoS Comput. Biol.* 2015 [cited 2024 Mar 3];11(2):e1003972. 10.1371/journal.pcbi.1003972 25654371 PMC4318577

[3] Crosswell LC Thornton JM : ELIXIR: A distributed infrastructure for European biological data. *Trends Biotechnol.* 2012 May 1 [cited 2024 Mar 3];30(5):241–242. 10.1016/j.tibtech.2012.02.002 22417641

[4] Harrow J Hancock J Blomberg N : ELIXIR-EXCELERATE: establishing Europe’s data infrastructure for the life science research of the future. *EMBO J.* 2021 Mar 15 [cited 2024 Mar 3];40(6):e107409. 10.15252/embj.2020107409 33565128 PMC7957415

[5] Orengo C Velankar S Wodak S : A community proposal to integrate structural bioinformatics activities in ELIXIR (3D-Bioinfo Community) [version 1; peer review: 1 approved, 3 approved with reservations]. *F1000Res.* 2020. 2020 Apr 22 [cited 2024 May 20];9(ELIXIR):278. 10.12688/f1000research.20559.1 32566135 PMC7284151

[6] Waterhouse RM Adam-Blondon AF Balech B : The ELIXIR Biodiversity Community: Understanding short- and long-term changes in biodiversity [version 2; peer review: 1 approved, 2 not approved]. *F1000Res.* 2023. 2023 May 15 [cited 2024 May 20];12(ELIXIR):499. 10.12688/f1000research.133724.2 PMC11179050 38882711

[7] Balech B Brennan L Carrillo de Santa Pau E : The future of food and nutrition in ELIXIR [version 1; peer review: 2 approved with reservations]. *F1000Res.* 2022. 2022 Aug 25 [cited 2024 May 20];11(ELIXIR):978. 10.12688/f1000research.51747.1

[8] Salgado D Béroud C Armean IM : The ELIXIR Human Copy Number Variations Community: building bioinformatics infrastructure for research [version 1; peer review: 2 approved]. *F1000Res.* 2020. 2020 Oct 13 [cited 2024 May 20];9(ELIXIR):1229. 10.12688/f1000research.24887.1 34367618 PMC8311797

[9] Davey NE Babu MM Blackledge M : An intrinsically disordered proteins community for ELIXIR [version 1; peer review: 2 approved]. *F1000Res.* 2019. 2019 Oct 15 [cited 2024 May 20];8(ELIXIR):1753. 10.12688/f1000research.20136.1 31824649 PMC6880265

[10] Robertsen EM Denise H Mitchell A : ELIXIR pilot action: Marine metagenomics – towards a domain specific set of sustainable services [version 1; peer review: 1 approved, 2 approved with reservations]. *F1000Res.* 2017. 2017 Jan 23 [cited 2024 May 20];6(ELIXIR):70. 10.12688/f1000research.10443.1 28620454 PMC5461914

[11] Steinbeck C Rijswijk M van Beirnaert C : The future of metabolomics in ELIXIR [version 2; peer review: 3 approved]. *F1000Res.* 2017. 2017 Oct 30 [cited 2024 May 20];6(ELIXIR):1649. 10.12688/f1000research.12342.2 29043062 PMC5627583

[12] Finn RD Balech B Burgin J : Establishing the ELIXIR Microbiome Community [version 1; peer review: 1 approved, 1 approved with reservations]. *F1000Res.* 2024. 2024 Jan 8 [cited 2024 May 20];13(ELIXIR):50. 10.12688/f1000research.144515.1 PMC12441670 40970218

[13] Pommier C Gruden K Junker A : ELIXIR Plant sciences 2020-2023 Roadmap. *F1000Res.* 2021. 2021 Feb 25 [cited 2024 May 20];10(ELIXIR):145 (document). 10.7490/f1000research.1118482.1

[14] Vizcaíno JA Walzer M Jiménez RC : A community proposal to integrate proteomics activities in ELIXIR [version 1; peer review: 2 approved]. *F1000Res.* 2017. 2017 Jun 13 [cited 2024 May 20];6(ELIXIR):875. 10.12688/f1000research.11751.1 28713550 PMC5499783

[15] D’Anna F Jareborg N Jetten M : A research data management (RDM) community for ELIXIR [version 1; peer review: 1 approved with reservations]. *F1000Res.* 2024. 2024 Mar 27 [cited 2024 May 20];13(ELIXIR):230. 10.12688/f1000research.146301.1 PMC11474151 39410979

[16] Czarnewski P Mahfouz A Calogero RA : Community-driven ELIXIR activities in single-cell omics [version 1; peer review: 2 approved with reservations]. *F1000Res.* 2022. 2022 Jul 29 [cited 2024 May 20];11(ELIXIR):869. 10.12688/f1000research.122312.1

[17] Martins dos Santos V Hancock JM Anton M : Systems Biology in ELIXIR: modelling in the spotlight [version 2; peer review: 1 approved, 2 approved with reservations]. *F1000Res.* 2022. 2022 Nov 7 [cited 2024 May 20];11(ELIXIR):1265. 10.12688/f1000research.126734.2 PMC9871403 36742342

[18] Martens M Stierum R Schymanski EL : ELIXIR and Toxicology: a community in development [version 2; peer review: 2 approved]. *F1000Res.* 2023. 2023 Oct 3 [cited 2024 May 20];10(ELIXIR):1129. 10.12688/f1000research.74502.2 37842337 PMC10568213

[19] Bacon J : *The Art of Community.* O’Reilly; Second ed. 2012.

[20] Acquaah M Amoako-Gyampah K Nyathi NQ : Measuring and Valuing Social Capital: A Systematic Review. 2014 [cited 2024 Jan 27]. Reference Source

[21] Castro LJ Martin C Lazarov G : Measuring outcomes and impact from the BioHackathon Europe. *BioHackrXiv Preprints [Preprint].* 2021 Aug 10 [cited 2024 Mar 3]. 10.37044/osf.io/3dxhg

[22] Shore LM Cleveland JN Sanchez D : Inclusive workplaces: A review and model. *Hum. Resour. Manag. Rev.* 2018 Jun [cited 2024 Jan 27] 1;28(2):176–189. 10.1016/j.hrmr.2017.07.003

[23] Gater C : ELIXIR Equal Opportunities Strategy. *F1000Res.* 2018 Aug 10 [cited 2024 Mar 3]. 10.7490/f1000research.1115874.1

[24] Likert R : A technique for the measurement of attitudes. *Arch. Psychol.* 1932;22:55. 140.

[25] Heil KF Garrard C : ELIXIR Communities Handbook 2024. *F1000Res.* 2024 Jan 22 [cited 2024 Mar 3];13(ELIXIR):80 (document). 10.7490/f1000research.1119695.1

[26] Woodley L Pratt K Sandström M : The CSCCE Skills Wheel – Five core competencies and 45 skills to describe the role of the community engagement manager in STEM. *Zenodo.* 2021 [cited 2024 Mar 3]. 10.5281/zenodo.4437293

[27] Heil KF : ELIXIR Communities Review - Process & Outcomes Form|ELIXIR 2019-2023 Programme. *Zenodo.* 2024 [cited 2024 Mar 3]. 10.5281/zenodo.10561817

[28] Wilkinson MD : *Comment: The FAIR Guiding Principles for scientific data management and stewardship.* Nature Publishing Group;2016 [cited 2024 May 15]. 10.1038/sdata.2016.18

[29] Galaxy Community: ELIXIR Galaxy Community Dashboard. [cited 2024 Mar 3]. Reference Source

[30] Galaxy Europe: Scientific research using & citing the European Galaxy server. [cited 2024 Jan 17]. Reference Source

[31] Papoutsoglou EA Faria D Arend D : Enabling reusability of plant phenomic datasets with MIAPPE 1.1. *New Phytol.* 2020 Jul 1 [cited 2024 Mar 3];227(1):260–273. 10.1111/nph.16544 32171029 PMC7317793

[32] Selby P Abbeloos R Backlund JE : BrAPI—an application programming interface for plant breeding applications. *Bioinformatics.* 2019 Oct 15 [cited 2024 Mar 3];35(20):4147–4155. 10.1093/bioinformatics/btz190 30903186 PMC6792114

[33] Mészáros B Hatos A Palopoli N : Minimum information guidelines for experiments structurally characterizing intrinsically disordered protein regions. *Nat. Methods.* 2023 Jul 3 [cited 2024 Mar 3];20(9):1291–1303. 10.1038/s41592-023-01915-x 37400558

[34] Dai C Füllgrabe A Pfeuffer J : A proteomics sample metadata representation for multiomics integration and big data analysis. *Nat. Commun.* 2021 Dec 1 [cited 2024 Mar 3];12(1):5854. 10.1038/s41467-021-26111-3 34615866 PMC8494749

[35] Claeys T Van Den Bossche T Perez-Riverol Y : lesSDRF is more: maximizing the value of proteomics data through streamlined metadata annotation. *Nat. Commun.* 2023 Dec 1 [cited 2024 Mar 3];14(1):6743. 10.1038/s41467-023-42543-5 37875519 PMC10598006

[36] Perez-Riverol Y Bai J Bandla C : The PRIDE database resources in 2022: a hub for mass spectrometry-based proteomics evidences. *Nucleic Acids Res.* 2022 Jan 7 [cited 2024 Apr 26];50(D1):D543–D552. 10.1093/nar/gkab1038 34723319 PMC8728295

