# Supplementary material for: Fostering and sustaining collaborative innovation: Insights from ELIXIR Europe's life science Communities
Source: F1000Res. 2025 Sep 9;14:ELIXIR-884. [Version 1] doi: 10.12688/f1000research.168288.1 (PMC12728338; doi:10.12688/f1000research.168288.1)
Supplement: Peer review history

# Reviewer report: Reviewer response for version 1

**DOI:** 10.5256/f1000research.185460.r413626

**Published:** 2025-12-23

**Authors:** Heidi Rehm

**Affiliations:** 1 Center for Genomic Medicine, Massachusetts General Hospital, Cambridge, MA, USA

This is a well-written and informative manuscript that describes ELIXIR Europe's life science Communities.  These Communities are designed to capture user needs across many domains of work in the life sciences and thereby inform the development of bioinformatics resources to support each domain. This paper describes the purpose and progress made in supporting these Communities. I have no criticisms or requests for revision.

Is the topic of the opinion article discussed accurately in the context of the current literature?

Yes

Are arguments sufficiently supported by evidence from the published literature?

Yes

Are all factual statements correct and adequately supported by citations?

Yes

Are the conclusions drawn balanced and justified on the basis of the presented arguments?

Yes

Reviewer Expertise:

human genetics and genomics; rare disease gene discovery and diagnosis; genomic resource development

I confirm that I have read this submission and believe that I have an appropriate level of expertise to confirm that it is of an acceptable scientific standard.

# Reviewer report: Reviewer response for version 1

**DOI:** 10.5256/f1000research.185460.r416799

**Published:** 2025-10-25

**Authors:** Amany Gouda-Vossos

**Affiliations:** 1 Australian Research Data Commons (ARDC), Melbourne, Australia

The article sets out to detail the insights and the benefits of the ELIXIR life science communities, that have been in operation for more than ten years (in operations since 2013) and provides 1. Insights into the ELIXIR framework, 2. Skill set of a community manager, and 3. Benefits of community with examples, including impacts. The goal of the article is to showcase the return on investment (ROI) from ELIXR’s community activities to Research Infrastructure (RI) developers, funders, and to provide a ‘playbook’ for others wanting to follow their model.

 Overall, the article was detailed and informative, providing any aspirational community developer some solid ground to follow. It was well written and easy to follow.

 The following suggestions below are minor and are for specific section where more clarity and details would be welcomed.  

**Introduction**

 Very minor detail – I found myself a little confused when I read that ELIXIR was operational in 2014, but had its ten-year anniversary in 2023 (does this mean it was established in 2013, but operational in 2014)?

**ELIXIR Communities framework**

 If possible, I suggest numbering the whole process throughout the text (e.g. 1. Proposal 2. Formal presentation with heads of nodes; 3. White paper…. (etc)) and corresponding that with Figure 1 to make it easier for the reader to follow.   

 The statement in page 6 “ *This process has evolved and matured over time with the formalisation and growth of community portfolio*” – As someone that would like to replicate this process, I would find it useful to read an example/description/reference of how this was managed (i.e. where did it start and how did it evolve?)  

**Table 1**

 Would it be possible to indicate which is ‘project’, which is a ‘Initiative’, and which is ‘other research infrastructure’ to make it easier for who are not familiar to follow (Maybe with symbols?)

*Co Leads and Governance*

- Who makes up the co-leads? Are they Researchers (at what level?)? PhD Students? How are they selected? What skills do they need?
- It would be great if a short explanation (or a diagram) of the relationship/hierarchical structure of the key stakeholders involved (i.e. Correct me if I’m wrong, but it appears like: ELIXIR hub heads---- *appoint*--->managers--- *who support-*-->Co-leads--- *that coordinate*--->members and activities)

**ELIXIR community case studies**

- Outside of describing establishing communities and providing much needed services – were there any outputs or metrics related to impact? ‘ *Contributions to Global Efforts*’ touches on this by stating that their server ‘had had more than 90 000 users since November 2018) and ‘ *Establishing community standards*’ describes the MIADE guidelines and how it is being implemented.
- Were there any other similar examples of impact for the others? 

Is the topic of the opinion article discussed accurately in the context of the current literature?

Yes

Are arguments sufficiently supported by evidence from the published literature?

Yes

Are all factual statements correct and adequately supported by citations?

Yes

Are the conclusions drawn balanced and justified on the basis of the presented arguments?

Yes

Reviewer Expertise:

I have expertise in biological sciences, and health and science education. I have over 10 years experience as a researcher and  have developed capability building training for various audiences for over 7 years.

I confirm that I have read this submission and believe that I have an appropriate level of expertise to confirm that it is of an acceptable scientific standard.
